# Germline variability and tumor expression level of ribosomal protein gene *RPL28* are associated with survival of metastatic colorectal cancer patients

**DOI:** 10.1038/s41598-019-49477-3

**Published:** 2019-09-10

**Authors:** Adrien Labriet, Éric Lévesque, Erika Cecchin, Elena De Mattia, Lyne Villeneuve, Michèle Rouleau, Derek Jonker, Félix Couture, David Simonyan, Eric P. Allain, Angela Buonadonna, Mario D’Andrea, Giuseppe Toffoli, Chantal Guillemette

**Affiliations:** 1 Pharmacogenomics Laboratory, Centre Hospitalier Universitaire de Québec (CHU de Québec) Research Center – Laval University and Faculty of Pharmacy, Laval University, Québec, Québec, Canada; 2 CHU de Québec Research Center and Faculty of Medicine, Laval University, Québec, Québec, Canada; 3 Clinical and Experimental Pharmacology, “Centro di Riferimento Oncologico”- National Cancer Institute, via Franco Gallini n. 2, 33081 Aviano, PN Italy; 4 Division of Medical Oncology, Department of Medicine, Ottawa Hospital, University of Ottawa, Ottawa, Ontario Canada; 5 Clinical and Evaluative Research Platform, CHU de Québec Research Center, Québec, Québec, Canada; 6 Medical Oncology Unit, “Centro di Riferimento Oncologico”- National Cancer Institute, via Franco Gallini n. 2, 33081 Aviano, PN Italy; 7 Medical Oncology Unit, “San Filippo Neri Hospital”, Via Giovanni Martinotti, 20, 00135 Rome, Italy; 8 Canada Research Chair in Pharmacogenomics, CHU de Québec Research Center, Québec, Québec, Canada

**Keywords:** Cancer genomics, Colorectal cancer

## Abstract

This study investigated the potential of single nucleotide polymorphisms as predictors of survival in two cohorts comprising 417 metastatic colorectal cancer (mCRC) patients treated with the FOLFIRI (folinic acid, 5-fluorouracil and irinotecan) regimen. The rs4806668G > T of the ribosomal protein gene *RPL28* was associated with shorter progression-free survival and overall survival by 5 and 9 months (*P* = 0.002), with hazard ratios of 3.36 (*P* < 0.001) and 3.07 (*P* = 0.002), respectively. The rs4806668T allele was associated with an increased *RPL28* expression in transverse normal colon tissues (n = 246, *P* = 0.007). *RPL28* expression was higher in colorectal tumors compared to paired normal tissues by up to 124% (*P* < 0.001) in three independent datasets. Metastatic cases with highest *RPL28* tumor expression had a reduced survival in two datasets (n = 88, *P* = 0.009 and n = 56, *P* = 0.009). High *RPL28* was further associated with changes in immunoglobulin and extracellular matrix pathways. Repression of RPL28 reduced proliferation by 1.4-fold to 5.6-fold (*P* < 0.05) in colon cancer HCT116 and HT-29 cells. Our findings suggest that the ribosomal RPL28 protein may influence mCRC outcome.

## Introduction

Metastatic colorectal cancer (mCRC) presents a 5-year relative survival just above 10%^1^. There are many treatment options for these patients including irinotecan-based chemotherapy. Particularly, the FOLFIRI regimen is composed of irinotecan (CPT-11) used in combination with 5-fluorouracil (5-FU) and folinic acid alone or with targeted therapies^2^. Both irinotecan and 5-FU are potent on actively replicating cancer cells. SN-38, the active metabolite of irinotecan, is an inhibitor of topoisomerase I (TOP1). It prevents DNA ligation by directly binding the TOP1-DNA complex, leading to replication arrest, double-strand breaks and cell death^3^. 5-FU is a pyrimidine analog that exerts its effect by inhibiting the thymidylate synthetase and DNA synthesis. 5-FU can also be incorporated into RNA during synthesis and interferes with protein synthesis^4^. The clinical response to FOLFIRI-based regimens is variable with dose-limiting toxicities occurring in a significant proportion of patients^5,6^. Several markers in pharmacokinetic pathways have been linked to severe toxicities. For instance, the UGT1A1*28 polymorphism was established as a predictive marker of severe neutropenia, explained by a decreased *UGT1A1* expression. Because this gene encodes the main hepatic enzyme responsible for SN-38 inactivation and elimination, reduced expression leads to greater exposure to SN-38 and an increased risk of severe neutropenia^7,8^. By contrast, clinical genetic-based evidence to predict therapeutic response of mCRC patients is limited^9^. Several genes were found to be associated with response to irinotecan and 5-FU *in vitro*, but their potential value as markers of treatment response has not been addressed in patients. These genes are related to several pathways such as detoxification of reactive oxygen species, cellular responses to stress, DNA damage recognition or DNA repair. In this study, we tested the hypothesis that genetic variability in these genes may be associated with survival of mCRC patients treated with FOLFIRI-based regimen.

## Results

### The RPL28 rs4806668G > T variant is associated with survival of mCRC patients

**Figure 1:**
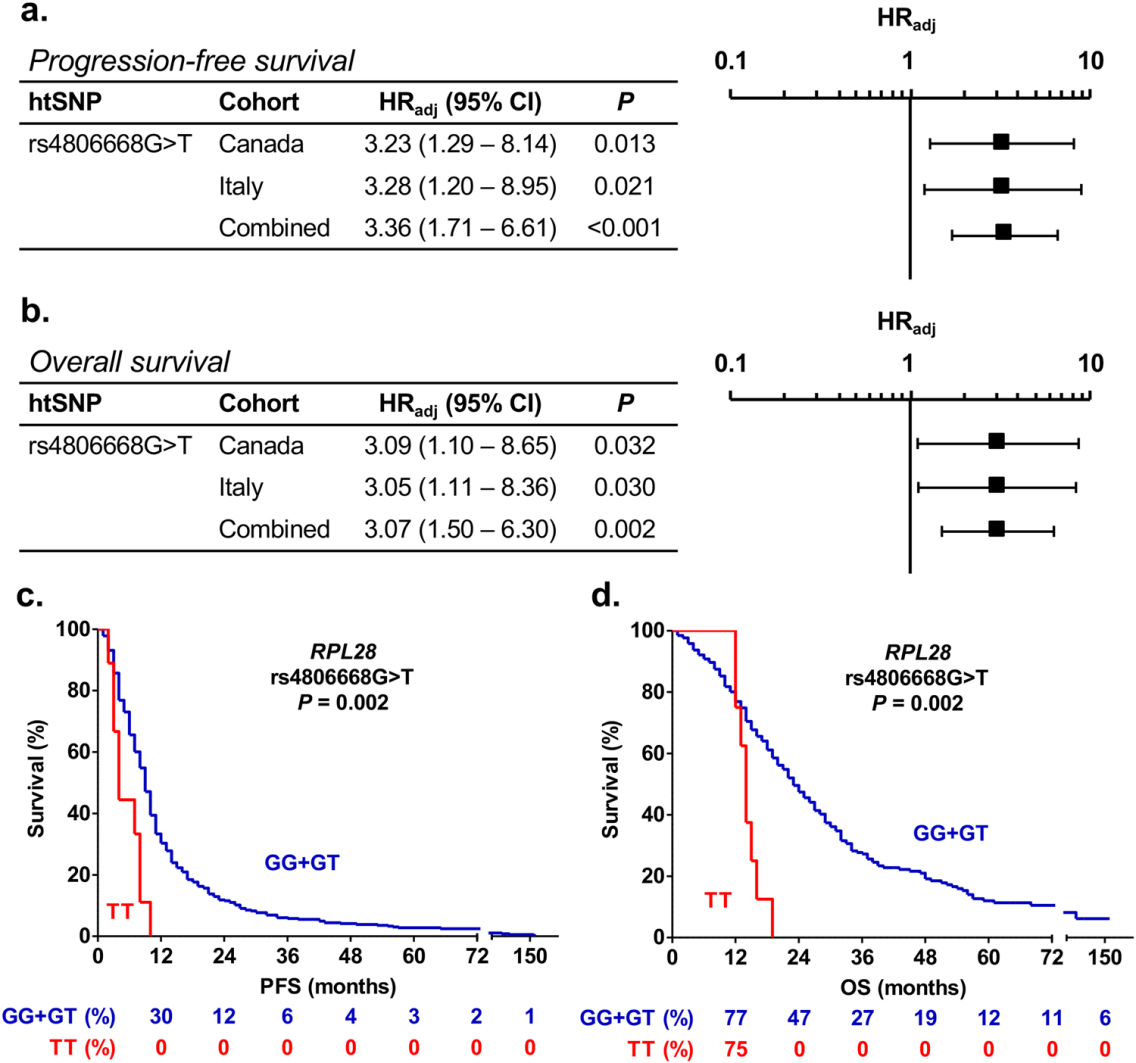
*RPL28* rs4806668G > T polymorphism is associated with survival  of mCRC patients treated with FOLFIRI. (**a**,**b**) Cox proportional hazards models adjusted for age and co-treatment (Canadian cohort, n = 167) and for age (Italian cohort, n = 250) showed association of rs4806668G > T with progression-free survival (PFS) and overall survival (OS) using a recessive genetic model. Tumor site did not have a statistically significant association with mCRC outcome. (**c**,**d**) Univariate Kaplan-Meier survival curves for PFS and OS according to rs4806668G > T genotype in combined cohorts. The percentage survival according to genotypes is shown under the graphs. HR_adj_, adjusted hazard ratio; htSNPs, haplotype-tagging single nucleotide polymorphisms; CI, confidence interval.

A screening of a total of 105 haplotype-tagging single nucleotide polymorphisms (htSNPs) covering the genetic variability of 17 candidate genes, which were previously associated with drug response *in vitro* or in patients (Additional file 1: Supplementary Tables 1 and 2) was performed in the discovery cohort comprising 167 mCRC Canadian patients. We identified 21 and 14 htSNPs associated (*P* < 0.1) with PFS and OS, respectively (Additional file 1: Supplementary Tables 3 and 4). In the replication cohort of 250 mCRC Italian patients, one of these htSNPs remained significantly associated (*P* < 0.05) with survival outcomes. The *RPL28* rs4806668G > T variant was associated with a reduced PFS in the Canadian cohort (hazard ratio (HR) = 3.23, *P* = 0.013), the Italian cohort (HR = 3.28, *P* = 0.021) and in combined cohorts (HR = 3.36, *P* < 0.001) using a recessive model (Fig. 1a). This marker was also linked to shorter OS with HR of 3.09 (*P* = 0.032), 3.05 (*P* = 0.030) and 3.07 (*P* = 0.002) in the Canadian, Italian and combined cohorts, respectively, using a recessive model (Fig. 1b). Overall, there were 9 carriers of rs4806668TT genotype. Kaplan-Meier analysis revealed that median PFS was reduced by 5 months for homozygous TT carriers compared to heterozygotes or non-carriers (9 months versus 4 months, *P* = 0.002, Fig. 1c), and OS reduced by 9 months (23 months versus 14 months, *P* = 0.002, Fig. 1d).

### The RPL28 rs4806668G > T variant affects RPL28 gene expression that is increased in tumor tissues

**Figure 2:**
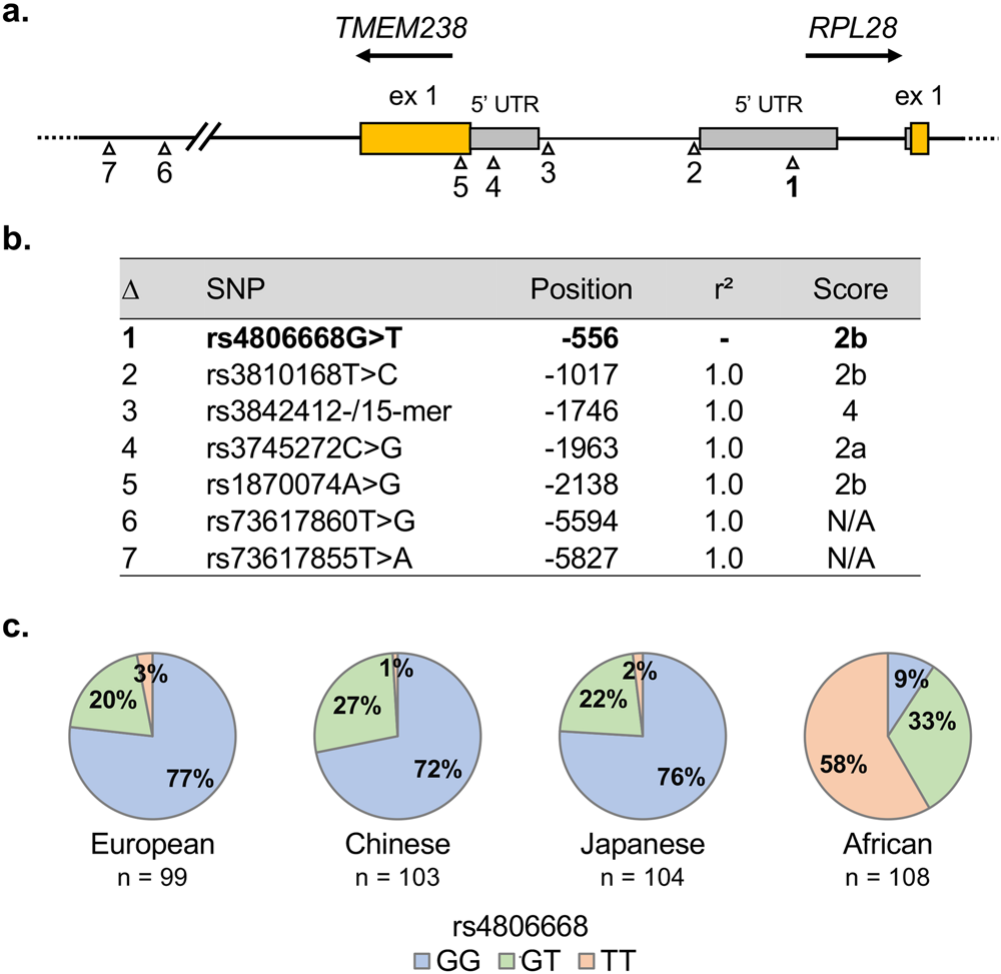
Rs4806668 is located in the promoter region of the *RPL28* locus and is linked to several other polymorphisms (SNPs). (**a**) Localization of the *RPL28* rs4806668G > T variant and its associated SNPs in strong linkage disequilibrium (r² > 0.80 in the European population). (**b**) Position of the rs4806668G > T marker and its linked SNPs relative to the translation start site of *RPL28*. Scores are from RegulomeDB and represent the probability for a SNP to be functional. N/A, not available. (**c**) Frequencies of *RPL28* rs4806668G > T among different ethnic groups (Ensembl GRCh38 release 91).

**Figure 3:**
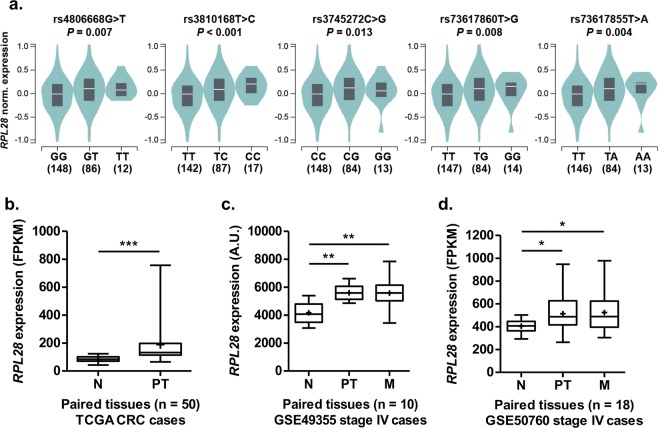
Relationship between *RPL28* variants, tissue type and gene expression. (**a**) *RPL28* rs4806668G > T and linked SNPs are associated with an increased gene expression in transverse colon tissues of healthy individuals from the GTEx cohort. (**b**) *RPL28* expression is higher in primary tumor relative to paired normal colorectal tissues (n = 50 pairs) from the TCGA cohort. (**c**) *RPL28* expression is higher in primary colorectal tumors and liver metastases relative to paired normal colorectal tissues (n = 10 pairs) from the GSE49355 dataset. (**d**) *RPL28* expression is higher in primary colorectal tumors and liver metastases relative to paired normal colorectal tissues (n = 18 pairs) from the GSE50760 dataset. A.U., arbitrary units; FPKM, fragments per kilobase million; N, normal tissue; PT, primary tumor tissue; M, liver metastases. *P < 0.05; **P < 0.01; ***P < 0.001.

The *RPL28* rs4806668G > T is located in the 5′-untranslated (5′-UTR) region of the *RPL28* locus and is in strong linkage disequilibrium in the CEU population (European population, LD with r² > 0.80) with six other SNPs, located upstream of the 5′-UTR (Fig. 2a,b). Four of these SNPs are predicted to affect transcription factor binding (score 2a or 2b) according to RegulomeDB (Fig. 2b). Genotypic frequencies of rs4806668G > T was found to be highly variable among ethnic groups with 1–3% of homozygous TT in populations of Asian and European origins and reaching 58% in the African population (Fig. 2c). To explore the potential impact of rs4806668G > T and its linked SNPs on *RPL28* gene expression, data from healthy donors of the GTEx project were used. An increased *RPL28* expression was observed for carriers of the variant allele rs4806668T (*P* = 0.007) in the transverse colon, as well as for variant alleles of SNPs in linkage disequilibrium (LD) with the rs4806668G > T (Fig. 3a). By contrast, expression of the nearby gene *TMEM238* was not affected. Data are summarized in Supplementary Table 5 (Additional file 1). In the TCGA cohort, *RPL28* expression was significantly higher by 124% (*P* < 0.001) in colon tumors compared to paired normal tissues (Fig. 3b). In mCRC cases from dataset GSE49355, *RPL28* expression was increased by 35% (*P* < 0.01) in primary tumors and by 34% (*P* < 0.01) in hepatic metastases compared to paired normal colon tissues (Fig. 3c). Similarly, in the GSE50760 dataset, *RPL28* expression was higher by 27% (*P* < 0.05) in primary tumors and by 30% (*P* < 0.05) in liver metastatic tissues, compared to paired normal tissues (Fig. 3d).

### High RPL28 expression is associated with reduced survival of mCRC patients, altered gene expression and affects colon cancer cells growth and survival *in vitro*

**Figure 4:**
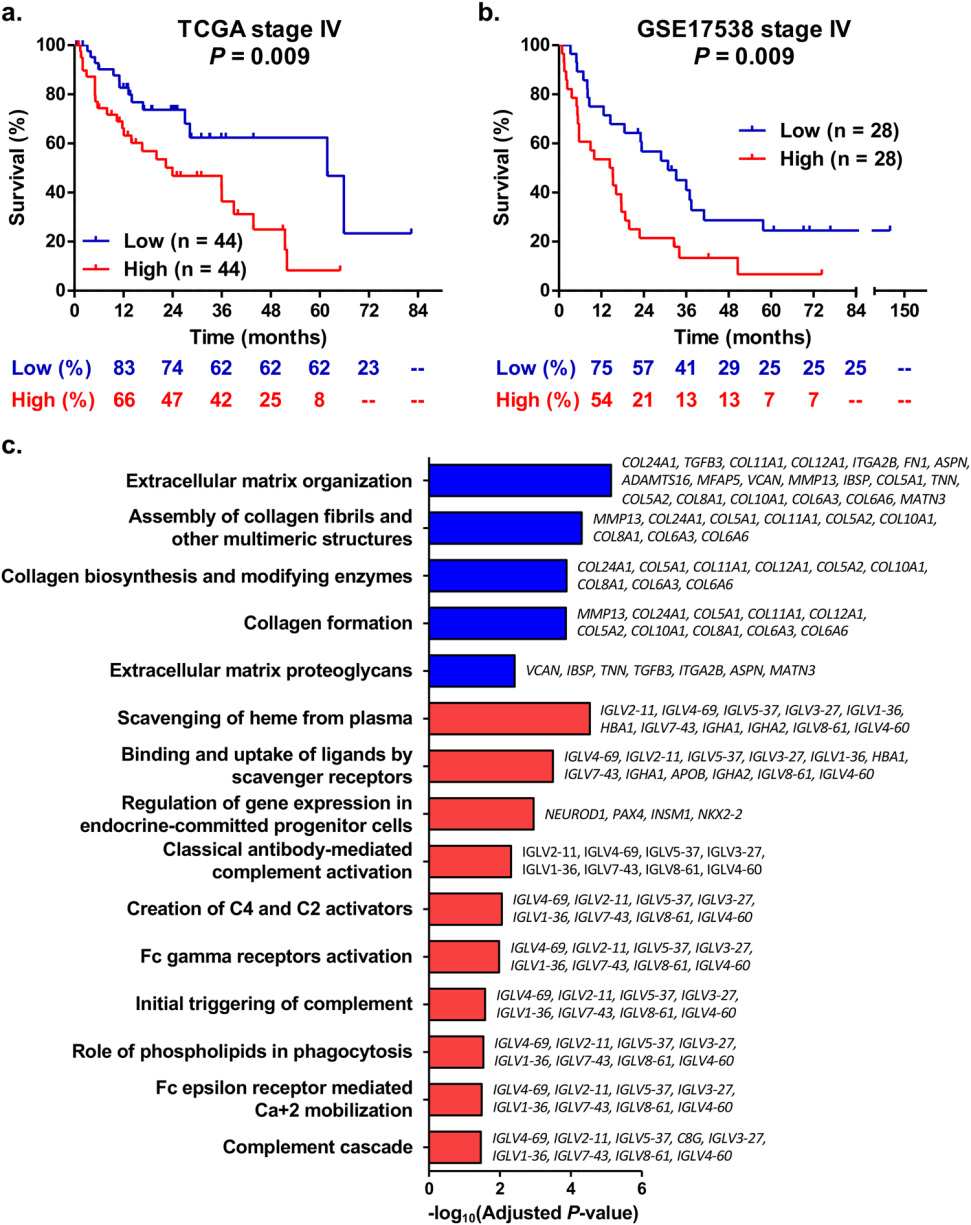
*RPL28* expression level in colorectal tumor tissues is associated with survival and changes in tumor transcriptome. (**a**) Kaplan-Meier curves for high and low *RPL28* expression groups (median separation) of stage IV mCRC individuals from the TCGA cohort (n = 88). The percentage survival according to *RPL28* expression group is shown under the graph. (**b**) Kaplan-Meier curves for high and low *RPL28* expression groups (median separation) of stage IV mCRC individuals from the GSE17538 dataset (n = 56). The percentage survival according to *RPL28* expression group is shown under the graph. The median and the optimal cut-off values of gene expression were highly similar (Additional file 1: Supplementary Fig. 1). (**c**) Significantly enriched pathways from differential gene expression in advanced stage IV mCRC cases with high *RPL28* expression compared to those with low *RPL28* expression from the TCGA cohort. Pathways in blue are enriched with down-regulated genes whereas those in red are enriched with up-regulated genes. Genes belonging to enriched pathways are shown on the right.

**Figure 5:**
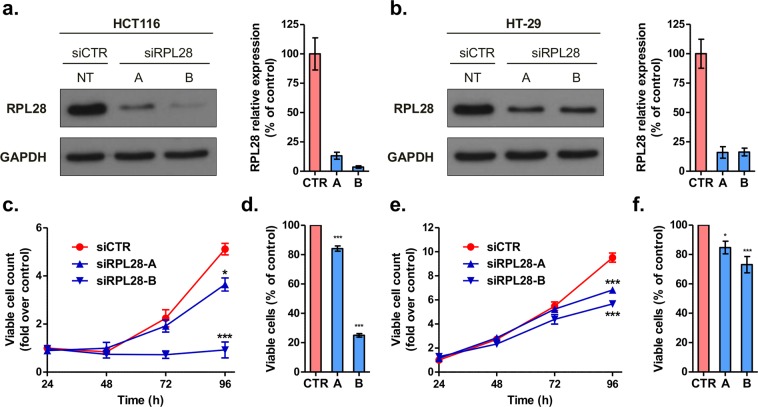
RPL28 expression levels influence proliferation of colorectal cancer cells. (**a**,**b**) Efficient knock-down of RPL28 protein expression in HCT116 and HT-29 exposed to specific siRNAs (siRPL28) relative to cells transfected with scrambled non-targeting (NT) negative control (siCTR). A representative cropped Western blot is shown for each cell line and a quantification of expression levels based on densitometry of blots from three independent experiments are shown. Full-length blots are shown in Additional file 1: Supplementary Fig. 2. (**c**–**f**) The knock-down of RPL28 impairs cell proliferation. Viable cells were monitored up to 96 hours after siRNA transfection in (**c**) HCT116 and (**e**) HT-29 (n = 3 in duplicate). Cell proliferation was measured by MTS assays 96 hours post-transfection in (**d**) HCT116 and (**f**) HT-29 (n ≥ 3 in triplicate). Data are presented as mean ± S.E.M. *P < 0.05; ***P < 0.001.

Based on the dichotomization at the median expression levels, mCRC patients from the TCGA cohort with high tumor expression of *RPL28* had a reduced survival compared to those with low levels (n = 88, *P* = 0.009; Fig. 4a). This observation was validated in mCRC cases from the dataset GSE17538 (n = 56, *P* = 0.009; Fig. 4b). Dichotomization at the optimal expression level cut-off also lead to significantly reduced survival for high *RPL28* expression groups, in both TCGA (*P* = 0.004) and GSE17538 (*P* < 0.001) datasets (Additional file 1: Supplementary Fig. 1). A differential gene expression and pathway enrichment analysis further indicated that over 804 genes were significantly altered (*FDR* < 0.05) in association with *RPL28* expression (high versus low, median separation) in cases of the TCGA cohort, including 285 down-regulated and 519 up-regulated genes (Additional file 2: Supplementary Table 6). From up-regulated genes, ten pathways related to immunoglobulins were enriched (adjusted *P*-value < 0.05) in cases with high *RPL28*, whereas five pathways related to extracellular matrix (ECM) or collagen were enriched from down-regulated genes (adjusted *P*-value < 0.05) (Fig. 4c). In these pathways, 17 genes comprised of mostly immunoglobulin genes were significantly upregulated (*FDR* < 0.05) in high *RPL28* cases, and a total of 20 genes, mainly collagen genes, were significantly down regulated (*FDR* < 0.05) (Fig. 4c). *In vitro* investigation further indicated significantly reduced proliferation by 1.4-fold to 5.6-fold (*P* < 0.05) and reduced viable cell ratios by 15 to 75% (*P* < 0.05) associated with knockdown of RPL28 in HCT116 and HT-29 colon cell lines (Fig. 5).

## Discussion

We report that the germline variant rs4806668G > T in *RPL28* and tumor expression of this gene are associated with survival of mCRC patients. The rs4806668TT genotype was associated with reduced PFS and OS in two independent cohorts of mCRC. This polymorphism was also associated with an increased expression of *RPL28* in colon tissue of healthy donors, and its expression was further increased in tumor tissues compared to paired normal tissues. In two other independent cohorts of mCRC, higher *RPL28* tumor expression level was associated with poor survival.

Our findings reveal that the *RPL28* rs4806668G > T is associated with PFS and OS in mCRC cases treated with first-line FOLFIRI regimen. FOLFIRI is one of the most common chemotherapy backbones for mCRC first-line treatment, however; only few studies investigated the potential of germline polymorphisms to predict its efficacy in several cohorts. Xu *et al*. identified the combination of UGT1A1*28 and UGT1A1*6 as predictive of reduced overall survival in two cohorts of Asian mCRC cases receiving irinotecan-based regimen in second- and third-line treatment^10^. A meta-analysis in Caucasians concluded that UGT1A1*28 cannot be considered as a reliable predictor of survival in CRC patients treated with irinotecan-based regimen^11^. Most studies including a discovery and a replication set focused on genes involved in targeted therapies – mainly bevacizumab – administered along with FOLFIRI, and reported markers of survival in several genes including *IL6*, *CD39*, *CXCR4* and *MKNK1*^12–16^. RPL28 is one of the 79 members of the ribosomal proteins (RPs) family. These proteins are constitutive components of the large subunit (RPL) and small subunit (RPS) of the ribosome primarily responsible for protein synthesis, which is greatly perturbed in tumor cells^17^. In addition to their role in ribosomal biogenesis and protein production, RPs possess ribosome-independent functions, especially in tumorigenesis, immune signaling and development^18^. This was notably demonstrated in breast, ovarian and pancreatic cancers as well as in osteosarcoma^19–22^. In CRC, a study showed that a higher level of the ribosomal RPS7 protein was associated with improved PFS and OS in 92 stage IV mCRC patients^23^. Another study of 200 CRC patients revealed a reduced OS associated with a higher level of RPS15A protein^24^. RPL28 upregulation was associated with reduced OS after azacytidine treatment in patients with myelodysplasia and related neoplasms^25^. These studies pointed toward potential functions of ribosome-free RPs that might engage an oncogenic role or suppressing tumor cell proliferation, depending on the RPs proteins involved^18^. A recent study suggested that *RPL28* was part of a hub of genes correlated to microsatellite instability status in CRC, indicating a potential role for this protein in defective DNA mismatch repair system associated with CRC^26^.

Various RPs are overexpressed in different cancer cells and are associated with the development and progression of malignant cancers. The *RPL28* htSNP rs4806668G > T and its two tightly linked SNPs (rs3810168T > C and rs3745272C > G) were associated with an increased *RPL28* expression in transverse colon of healthy individuals and were predicted to be functional based on RegulomeDB data, in support of a causal link with at least one of these SNPs. In the transverse colon, *RPL28* expression level appeared similar for GT and TT carriers and higher than in GG carriers of rs4806668. However, an allele dose effect was observed for the linked rs3810168T > C, rs73617860T > G and rs73617855T > A, with higher expression for minor homozygous carriers, in line with the recessive model of the association in our cohorts. An upregulation of *RPL28* was further noted in tumors compared to paired normal tissues suggesting an oncogenic role of this ribosomal protein in CRC. In support of this notion, repression of RPL28 was shown to significantly impair cell proliferation of HCT116 and HT-29 colon cancer cell models, suggesting that *RPL28* higher expression may be associated to a more aggressive phenotype. Knockdown of other RPs such as RPL9 and RPS24 suppressed colon carcinoma cell growth *in vitro* and human CRC xenografts in nude mice^27,28^. Similarly, overexpression of RPS7 led to reduced proliferation of a panel of 4 CRC cell lines including HCT116 and HT-29, suggested to be mediated through repression of HIF-1α and glycolysis^23^.

Our analysis of transcriptomic changes associated with high *RPL28* tumor expression in mCRC patients suggested an upregulation of several genes of immunoglobulins, proposing the involvement of the complement system. Although the complement is recognized as an important actor of the immune system that contributes to the destruction of cancer cells, recent data have indicated a tumor-promoting role^29,30^. Several studies support that malignant cells efficiently activate complement and that metastatic pathways are triggered by imbalanced complement activation and inflammation^31^. Recently, RPL28 expression was inversely associated to viral peptide presentation by the major histocompatibility complex class I, suggesting a potential role of this ribosomal protein in the immune system processing^32^. Immunosuppressive properties were proposed for RPS19, also increased in colon cancer cells and released from apoptotic tumor cells^33^. It was further suggested that RPS19 immunosuppressive role in cancer involved its interaction with the complement receptor C5aR1 (CD88), promoting tumor growth in a transgenic model of breast cancer^34^. Another study reported increased cell invasiveness in response to C5a-induced secretion of metalloproteinases from C5aR1-expressing cancer cells and degradation of extracellular matrix (ECM), known to contribute to neoplastic progression^35,36^. Indeed, we observed a down-expression of several ECM genes in high compared to low *RPL28* expressing primary tumors. A role for ECM components such as collagen, fibronectin or matrix metalloproteinases was clearly established in cancer development and metastatic spread^37^. A down-regulation of genes related to ECM adhesion and remodeling was also observed between hepatic CRC metastases and primary colorectal tumors^38^. As a lower adhesion is thought to favor metastasis and dissemination, a down-regulation of ECM related genes in the high *RPL28*/low survival group of patients might be the sign of a higher susceptibility for cancer cells to spread more efficiently and quickly from the primary tumor^39^. Overall, it may be envisioned that the changes in gene expression associated with *RPL28* activate cancer-related signaling pathways and exert tumor-promoting activities in mCRC patients.

A limitation of this study was that we did not apply correction for multiple testing in the polymorphism association study due to its exploratory nature. However, we studied two independent cohorts to corroborate initial findings, limiting the possibility of false-positive associations. We also included multiple datasets, including the large GTEx and TCGA cohorts, to study associations with gene expression. We acknowledge that our study design comprising only FOLFIRI-treated patients did not allow to clarify the predictive value of the positive marker and that further investigations are needed to explain the molecular mechanisms by which RPL28 affects survival and its possible interplay with ECM and immunoglobulin pathways. Given the high frequency of the rs4806668TT genotype in Africans, it would be of interest to evaluate further the association of the rs4806668G > T with clinical outcome in this population. Finally, the lack of data regarding microsatellite instability in our datasets precluded further analysis of its influence on mCRC outcome in relation to RPL28 status. One further limitation was our inability to account for treatment status in relation to *RPL28* expression in the public datasets^40^.

In conclusion, we reported the germline variant *RPL28* rs4806668G > T, associated with increased gene expression, as a novel marker of survival  of mCRC patients treated with the FOLFIRI regimen. We further established that high *RPL28* tumor expression is linked to  a reduced survival of mCRC patients, possibly through an enhanced tumor growth and a remodelling of the expression of ECM and immunoglobulin pathways. This study highlights a potential role of the ribosomal protein RPL28 in colon tumorigenesis and as a possible prognostic survival marker of mCRC patients.

## Methods

### Characteristics of cohorts

Two independent cohorts of mCRC patients were included in the study. They were previously described in details, including the study eligibility criteria and genomic DNA isolation procedure^41,42^. Briefly, the discovery cohort  consisted of 167 patients all treated with FOLFIRI-based regimen recruited in three centers of Eastern Canada from 2003 to 2012. These patients received FOLFIRI and 75 were also co-treated with bevacizumab or an experimental drug. The replication cohort enrolled 250 mCRC patients recruited in 13 centers of Northeastern Italy between 2002 and 2005 all treated with FOLFIRI. Clinical outcomes for this study were progression-free survival (PFS, defined as the time between the beginning of FOLFIRI treatment and the first evidence of disease progression, including death, or the last follow-up) and overall survival (OS, defined as the time between the beginning of FOLFIRI treatment and death from any cause). Characteristics of both cohorts are summarized in Table 1. Ethnicity was self-reported with the vast majority identified as Caucasian or White. All patients provided written informed consent and the research protocol was approved by the local ethics committees (CHU de Québec ethics committee for the Canadian cohort and Comitato Etico Indipendente - Centro di Riferimento Oncologico di Aviano for the Italian cohort). This study was conducted in accordance with the declaration of Helsinki.

**Table 1:** Characteristics of the discovery and replication mCRC cohorts.

Characteristics	Canadian cohort		Italian cohort	
	N = 167	%	N = 250	%
Gender				
Male	110	66	162	65
Female	57	34	88	35
Age (years)				
Mean	61.5	—	60.6	—
Standard Deviation	10.2	—	10.3	—
Range	29–86	—	26–75	—
FOLFIRI	167	100	250	100
Co-treatment	75	44.9	0	0
Bevacizumab	69	92.0	0	0
Other drug	6	8.0	0	0
Clinical outcomes (median in months)				
Progression-free survival	11	—	7	—
Overall survival	24	—	15	—

### Genetic analysis

Single Nucleotide Polymorphisms (SNPs) with a minor allele frequency higher than 5% were identified from the European (CEU) population of the International HapMap project for the selected candidate genes (Additional file 1: Supplementary Table 1). Haplotype-tagging SNPs (htSNPs) in high linkage disequilibrium (LD, r² > 0.80) in the CEU population were selected with Haploview v4.2 (Broad Institute, Cambridge, MA, United States)^43^. A complete list of htSNPs and their associated SNPs is provided as supplementary data (Additional file 1: Supplementary Table 2). All htSNPs were genotyped using the Sequenom iPLEX matrix-assisted laser desorption/ionization time-of-flight mass spectrometry technology (Sequenom, San Diego, CA, United States). Genotypic frequencies and LD data were retrieved through Ensembl GRCh38 release 91 - December 2017 (https://www.ensembl.org/index.html) for European (CEU), Chinese (CHB), Japanese (JPT) and African (YRI) populations. RegulomeDB v1.1 was used to investigate whether polymorphisms may have a functional impact^44^.

### Statistical analysis

Hardy-Weinberg equilibrium was verified using PLINK v1.07^45^ and SNPs deviating from the equilibrium (*P* < 0.05) were excluded from further analysis. Associations between htSNPs and clinical outcomes were tested using Cox proportional hazards model adjusted for age and co-treatment in the Canadian and combined cohorts and for age in the Italian cohort. Additive, dominant and recessive models were fitted independently for each htSNP. Analyses were carried out using SAS version 9.4 (SAS Institute Inc., Cary, NC, United States) and R v3.2.2 Survival package. Genetic variants were initially tested in the Canadian cohort and those with a *P*-value < 0.1 were genotyped in the Italian cohort. A marker was considered validated when associated to survival using the same genetic model in both cohorts (*P* < 0.05). Kaplan-Meier curves and log-rank test *P*-values were estimated with GraphPad Prism 5 (GraphPad Software Inc., La Jolla, CA, United States). Groups based on gene expression for Kaplan-Meier analysis were separated at the median or at the optimal cut-off values of gene expression, defined as the point with the most significant log-rank test split determined by Cutoff Finder version 2.1^46^.

### Expression data

Normalized gene expression and *P*-value data from the Genotype-Tissue Expression (GTEx) project (Analysis Release V7) were obtained through the GTEx portal (https://www.gtexportal.org/home/) on May 29, 2019. The Cancer Genome Atlas (TCGA, https://cancergenome.nih.gov/) was used to obtain data of Colon Adenocarcinoma (TCGA-COAD) and Rectum Adenocarcinoma (TCGA-READ) projects, accessed through the Genomic Data Commons Data Portal (https://portal.gdc.cancer.gov/) on October 2, 2018. HTseq counts (raw counts) for stage IV TCGA-COAD and TCGA-READ individuals were retrieved with TCGAbiolinks package (version 2.9.5) in R and processed for TMM normalization with edgeR package (version 3.22.3). Differential gene expression between individuals with high and low *RPL28* expression levels based on the median separation was then performed using edgeR package and exact test method. Ensembl gene ID were converted into gene symbols with g:Profiler^47^. Pathway enrichment analysis with significantly differentially expressed genes (*FDR* < 0.05) and log2(FC) lower than −1.2 or higher than 1.2 was performed using Reactome 2016 library in Enrichr^48,49^. Expression and clinical data publicly available for dataset GSE49355, GSE50760 and GSE17538 were obtained through GEO database with GEOquery package (version 2.48.0)^38,50,51^. Groups were compared using a two-tailed paired t-test or a one-way ANOVA followed by a *post-hoc* Dunnett’s test in GraphPad Prism 5 software.

### *In vitro* experiments

HCT116 and HT-29 cell lines were obtained from the American Type Culture Collection (Rockville, MD, United States) and grown as recommended. Cells were passaged less than 15 times between thawing and completion of experiments. Cells were reverse-transfected in 96-well or 24-well plates with a final concentration of 20 nM siRNA (Dharmacon, Chicago, IL, USA) using DharmaFECT2 Transfection Reagent as per the manufacturer’s recommendations. Two siRNA targeting RPL28 (D-011145-02 or siRPL28-A and D-011145-03 or siRPL28-B) and a scrambled non-targeting negative control (D-001206-14 further referred as siCTR) were tested. Viable cells were counted at 24 h, 48 h, 72 h and 96 h post-transfection using trypan blue and an automated cell counter (Bio-Rad, Mississauga, ON, Canada). For each time point, fold over control was calculated by dividing the viable cell count by the viable cell count of siCTR at 24 h. Experiments were carried out three independent times in duplicate. Viable cell proliferation at 96 h was assessed by an MTS assay (CellTiter 96 AQueous One Solution Cell Proliferation Assay, Promega, Madison, WI, United States) followed by measurement of absorbance at 490 nm. Viable cell proliferation was expressed as the ratio of absorbance for each condition over absorbance for siCTR. At least three independent experiments were performed in triplicate. At 96 h, conditions were compared using a one-way ANOVA followed by a *post-hoc* Dunnett’s test in GraphPad Prism 5 software. For protein expression, cells were washed with PBS 72 hours post-transfection and harvested in lysis buffer [0.05 M Tris-HCl pH 7.4, 0.15 M NaCl, 1% (w/v) Igepal CA-630 (Sigma-Aldrich), 1 mM dithiothreitol, and Complete protease inhibitor cocktail (Roche, Laval, QC, Canada)], lysed on a rotation unit for 30 min and cleared by centrifugation for 15 min at 13000 *g*. Cell lysates (5 μg) were resolved on a 12% polyacrylamide gel and transferred to a nitrocellulose membrane. RPL28 protein was detected using a rabbit polyclonal anti-RPL28 antibody (Proteintech, Group, 16649-1-AP, Rosemont, IL, United States) and GAPDH, used as a loading control, with a mouse monoclonal anti-GAPDH antibody (G8795, Sigma-Aldrich). RPL28 protein levels were determined by densitometry scanning of band intensity and normalized by the corresponding GAPDH band intensity on immunoblots using ImageJ (https://imagej.nih.gov/ij/). Experiments were carried out three independent times.

## Data Availability

The datasets used and analysed during the current study are available from the corresponding author on reasonable request.

## Supplementary information

Additional_File_1

Additional_File_2
