# Supplementary material for: Germline variability and tumor expression level of ribosomal protein gene RPL28 are associated with survival of metastatic colorectal cancer patients
Source: Sci Rep. 2019 Sep 10;9:13008. doi: 10.1038/s41598-019-49477-3 (PMC6736932; doi:10.1038/s41598-019-49477-3)
Supplement: Supplementary file 1 — Additional_File_1 [file 41598_2019_49477_MOESM1_ESM.docx]

**Germline variability and tumor expression level of ribosomal protein gene *RPL28* are associated with survival of metastatic colorectal cancer patients**

Adrien Labriet, Éric Lévesque, Erika Cecchin, Elena De Mattia, Lyne Villeneuve, Michèle Rouleau, Derek Jonker, Félix Couture, David Simonyan, Eric P. Allain, Angela Buonadonna, Mario D’Andrea, Giuseppe Toffoli and Chantal Guillemette

**Supplementary Material**


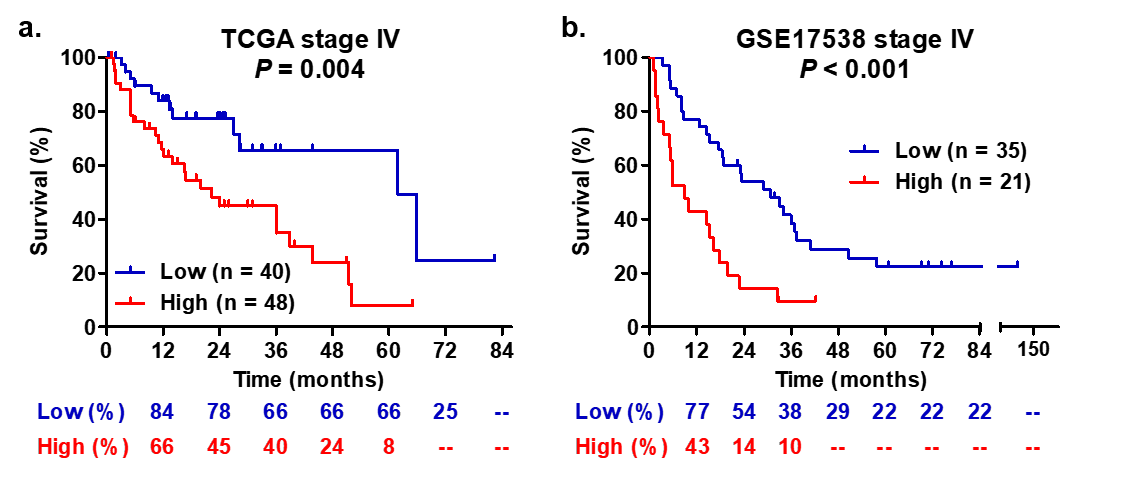


**Supplementary Figure 1**. *RPL28* expression level in colorectal tumor tissues is associated with survival. **A.** Kaplan-Meier curves for high and low *RPL28* expression groups (dichotomization at the optimal cut-off of expression levels) of stage IV mCRC individuals from the TCGA cohort (n = 88). The percentage survival according to *RPL28* expression group is shown under the graph. **B.** Kaplan-Meier curves for high and low *RPL28* expression groups (dichotomization at the optimal cut-off of expression levels) of stage IV mCRC individuals from the GSE17538 dataset (n = 56). The percentage survival according to *RPL28* expression group is shown under the graph. This figure is related to Figure 4a and 4b.

**
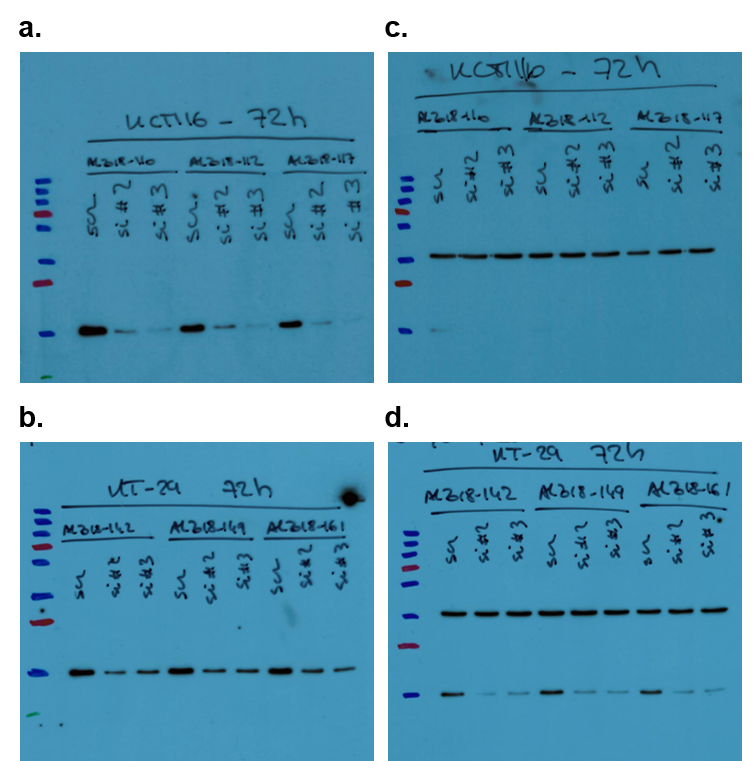
**

**Supplementary Figure 2.** Full-length blots related to Figure 5. Western blots for the detection of RPL28 in HCT116 (a) and HT-29 (b) are shown. GAPDH was detected on the same blots for HCT116 (c) and HT-29 (d). scr, siCTR; si#2, siRPL28-A; si#3, siRPL28-B.

**Supplementary Table 1**. Relation between selected genes and irinotecan and/or 5-fluorouracil response.

| **Gene** | **Gene full-name** | **Link with irinotecan and/or 5-FU** | **Ref.** |
| --- | --- | --- | --- |
| *CDC45* | Cell division cycle 45 | Involved in TOP1-apoptosis pathway | [1](#_ENREF_1) |
| *MAPK9* | Mitogen-activated protein kinase 9 | Silencing enhances SN-38 effect *in vitro.* | [2](#_ENREF_2) |
| *PARP1* | Poly(ADP-ribose) polymerase 1 | PARP1 inhibition increases sensitivity of colon cancer cell lines to irinotecan. Involved in the repair of TOP1-induced DNA damages. | [3](#_ENREF_3) |
| *SART1* | U4/U6.U5 tri-snRNP-associated protein 1 | Silencing increases sensitivity to SN-38 and 5-FU *in vitro.* | [2](#_ENREF_2) |
| *ACTL6A* | Actin like 6A | Down-regulated in irinotecan-resistant cells. | [4](#_ENREF_4) |
| *CBX3* | Chromobox 3 | Down-regulated in irinotecan-resistant cells. | [5](#_ENREF_5) |
| *CFL1* | Cofilin 1 | Differentially expressed in irinotecan-resistant cells. | [4](#_ENREF_4),[5](#_ENREF_5) |
| *CRYAB* | Crystallin alpha B | Up-regulated in irinotecan-resistant cells. | [4](#_ENREF_4) |
| *NME1-2* | NME/NM23 nucleoside diphosphate kinase 1-2 | Differentially expressed in irinotecan-resistant cells. | [4](#_ENREF_4),[5](#_ENREF_5) |
| *PRDX2* | Peroxiredoxin 2 | Down-regulated in irinotecan-resistant cells. | [5](#_ENREF_5) |
| *PRDX3* | Peroxiredoxin 3 | Up-regulated in irinotecan-resistant cells. | [5](#_ENREF_5) |
| *PRDX4* | Peroxiredoxin 4 | Differentially expressed in irinotecan-resistant cells. | [4](#_ENREF_4),[5](#_ENREF_5) |
| *CAVIN1 (PTRF)* | Caveolae associated protein 1 | Silencing enhances the toxicity of SN-38 and 5-FU *in vitro.* | [2](#_ENREF_2) |
| *RPL28* | Ribosomal protein L28 | Silencing enhances the toxicity of SN-38 and 5-FU *in vitro*. | [2](#_ENREF_2) |
| *TDP1* | Tyrosyl-DNA phosphodiesterase 1 | *TDP1* IVS12+79 associated with response to irinotecan-based therapy in advanced colorectal patients. Involved in the removal of TOP1-DNA adducts. | [1](#_ENREF_1),[6](#_ENREF_6) |
| *GSTP1* | Glutathione S-transferase pi 1 | *GSTP1* Ile105Val was associated with survival and toxicities in mCRC patients. | [7](#_ENREF_7),[8](#_ENREF_8) |
| *TIMP1* | TIMP metallopeptidase inhibitor 1 | TIMP1 levels were associated with survival in mCRC patients receiving combination regimen including irinotecan and 5-FU. | [9](#_ENREF_9),[10](#_ENREF_10) |

**Supplementary Table 2**. Haplotype-tagging single nucleotide polymorphisms (htSNPs) and their associated SNPs.

| **Genes** | **htSNPs** | **Associated SNPs** |
| --- | --- | --- |
| *CDC45* | rs4141528 | rs4141527 rs5748228 rs5748227 rs5748222 rs5746741 rs5748241 rs77362076 rs5746756 rs5748212 rs150190062 rs5746757 rs5748211 rs143338547 rs79114187 rs2283656 rs13447273 rs117554245 rs13447294 rs13447302 rs12628900  rs79771248 rs45596741 |
|  | rs5993650 | rs5992403 rs5993649 rs5748231 rs35880706 rs2238772 rs59690810 rs5748226 rs2073758 rs5993651 rs5993652 rs5993648 rs11704146 rs739369 rs9618585 rs756657 rs756658  rs5746742 rs4493360 rs2073759 rs2072055 rs9606025 rs5993647 rs11289637 rs5748219 rs13447232 rs5993654 rs8137899 rs2097600 rs9604966 rs1002111 rs5993657 rs9606024 rs5746738 rs2269722 rs2073761 rs8136840 rs36124072 rs12484607 rs2073727 rs2073737 rs2073736 rs9606042 rs7292006 rs1057457 rs9606043 rs2073730 rs2073731 rs5993661 rs137937633 rs2283655 rs2073735 rs2073733 rs2006242 rs9606048 rs739370 rs9606017 |
|  | rs737858 | rs5746748 rs5746749 rs5748238 rs5746744 rs5746743 rs1547931 rs2238771 rs5748246 rs5748218 rs5748251 rs5748217 rs5748216 rs5746740 rs7364291 rs5748215 rs2269721 rs5748254 rs1548359 rs2904558 rs756649 rs712979 rs9604965 rs5748259 rs2871030 rs2871031 rs5748260 |
|  | rs9618586 | rs5748243 rs7289378 |
|  | rs2073736 | rs2073737 rs9606042 rs9606043 rs8136840 rs2073761 rs5993661 rs5993657 rs2283655 rs1002111 rs2097600 rs2073735 rs5993654 rs2073733 rs13447232 rs11289637 rs9606046 rs2073759 rs5992413 rs5993665 rs9618585 rs11704146 rs5993652 rs5993651 rs9606048 rs739370 rs59690810 rs2238772 rs5993650 rs5992403 rs5993649 rs5748231 rs35880706 rs5748226 rs2073758 rs5993648 rs739369 rs756657 rs756658 rs5746742 rs4493360 rs2072055  rs9606025 rs5993647 rs5748219 rs8137899 rs9604966 |
|  | rs2073732 | rs5746758 rs5748252 rs5748240 |
| *MAPK9* | rs7724543 | rs11310451 rs17080136 rs33928286 rs7730064 rs7730091 rs13162206 rs17627593 rs6874413 rs6896513 rs6875134 rs11960340 rs34428073 rs35203283 rs6601104 rs9605 rs7710896 rs56131100 rs13178637 rs61478999 |
|  | rs6877489 | rs35394934 rs17627536 rs35759987 rs55756747 rs6894217 rs1127580 rs62406991 rs3099063 rs3111525 |
|  | rs4362908 | rs7726664 rs4481314 rs35081878 rs11955223 rs62404860 rs1127575 rs58528869 |
|  | rs11956696 | rs1035413 rs6898872 rs6898670 rs33979712 rs17683933 rs35588458 rs35254089 rs35691219 rs4601008 rs4639174 rs35624369 rs36010045 rs73811323 rs73811322 rs73811321 rs56320879 rs115200228 rs77310082 rs56264608 rs74399328  rs73338764 rs79172932 |
|  | rs11743530 | -- |
|  | rs3812067 | rs7715540 rs6601106 rs6872544 rs35777469 rs56658635 rs6601105 rs13169028 rs7729984 |
|  | rs12522876 | rs12518159 rs57403593 rs10052124 rs12519649 rs2112593 |
|  | rs7445806 | rs56333381 rs4634313 rs13435 rs34715282 rs6601103 |
|  | rs3812066 | rs35977675 rs72811017 rs72811018 rs113747850 rs888923 rs72811021 rs72811022 rs76752307 rs77249190 rs72811024 rs34876832 rs140492570 rs17628029 |
|  | rs6896347 | rs11249687 |
|  | rs6867398 | rs62404891 rs7720684 rs55913314 rs66800744 rs140356894 rs62404890 rs730688 rs7708873 rs7722861 |
|  | rs3111515 | -- |
|  | rs6895740 | rs3111537 |
|  | rs4147385 | -- |
|  | rs4700730 | rs11249689 rs7721150 rs6879043 |
|  | rs7714207 | -- |
|  | rs13185784 | rs17627880  rs17627815  rs35067749  rs6893005 |
|  | rs6868333 | rs33943370 rs6886915 rs732904 rs731918 rs13178637 |
|  | rs7713083 | -- |
| *PARP1* | rs1805415 | rs3219065 rs2117114 rs907190 rs3219082 rs2255403 rs932002  rs2027440 rs1805411 rs3219104 rs2377312 rs907191 rs907192  rs907193 rs2048425 rs34742465 rs1136410 rs2793381 rs2793658 rs2271349 rs11378196 rs34917861 rs146042055  rs1805404 rs752307 rs752309 rs1891107 rs1891108 rs2695243  rs2695242 rs892348 rs907187 rs12143101 rs56033994 rs2793379 rs1317170 rs2249731 rs76887998 rs138994074  rs2570367 rs9943081 rs4653729 rs4653728 rs1433574 |
|  | rs3219110 | -- |
|  | rs1805410 | rs2271345 rs3219095 rs3219049 rs71801596 |
|  | rs8679 | rs2271347 rs61835377 rs555462858 rs2793383 rs1805403 rs74973162 |
|  | rs3754376 | rs3835704 rs3219112 rs3219090 rs3219119 rs747657 rs1805414 rs2027439 rs2048424 rs2793382 rs2377313 rs10631977 rs59275599 rs2793380 rs2666428 rs2793657 rs1417765 rs2048426 rs144361550 rs1341336 rs11366269 rs2793378 rs1104893 rs2793377 rs35170928 rs2377013 rs11304925 rs2249844 rs1397550 rs1828446 rs1828445 rs2793654 rs2666427 rs1865222 rs1865221 rs1865220 rs2695235 rs2793655 rs2793376 rs2695236 rs575478286 rs2695237 rs9426568 rs35242305 rs2695238 rs1341333 rs1341334 rs1341335 rs2695239 rs2570368 rs2570369 rs2136875 rs2695240 rs2570370 rs1858548 rs35380305 rs1858549 rs1858550 |
|  | rs12568297 | -- |
|  | rs6668722 | rs142801988 rs146044150 rs10915984 rs141794185 rs367797484 rs12119288 |
|  | rs3219123 | rs78087823 |
|  | rs6668851 | rs6702917 rs6426551 rs7517174 |
|  | rs1805401 | -- |
|  | rs1805405 | rs2280712 rs1002153 rs1000033 rs2136876 rs1073991 rs3219043 rs4653445 rs12240196 rs2293464 rs4653731 rs3046773 rs7548007 rs7542788 rs12068649 rs3219053 rs10799349 rs1805408 rs3219058 rs6701634 rs3219031 rs3219027 rs3219073 rs3219074 rs3219023 rs3219022 rs3754370 rs2271343 rs3768347 rs3768346 rs732284 rs7522351 rs7525191 rs4653732 rs4653733 rs60698376 rs4653734 rs7531668 rs2077197 rs7527192 rs59672299 rs1109032 rs3754375 rs6665208 rs532472660 rs141615498 rs4653735 rs6679573 rs10915987 rs77173384 rs28407557 rs3219115 rs12068460 rs12025487 rs752308 rs747658 rs747659 rs6681537 rs6664761 rs2282400 rs6675427  rs6675327 rs6661762 rs1991865 rs12092726 rs6700864 rs878367 |
|  | rs3219142 | rs68193591 |
| *SART1* | rs688862 | rs556643 rs552130 rs550435 rs1151505 rs660118 rs12797319 rs11606402 rs1192168 rs642293 rs56239996 rs55704213 rs35239693 rs593525 rs491973 rs501353 rs11227368 rs55928757 rs1211729 rs470192 rs56154109 rs649000 rs542332 rs1308733 rs679581 rs493899 rs28578718 rs551659 rs142280625 rs4994056 rs503524 rs659982 rs661335 rs566590 rs624273 rs1786175 rs606978 rs538954 rs1151512 rs141871632 rs531612 rs12792888 rs528736 rs7105628 rs56152520 rs11227354 rs1204649 rs617791 rs3016868 rs12361552 rs674363 rs613924 rs14157 rs12794370 rs147010511 rs590318 rs10609752 rs694156 rs61893932 rs655744 rs632578 |
|  | rs7952056 | rs60109456 rs11227370 rs7939253 rs735942 rs35833455 rs10791835 rs7934543 rs34844246 rs55947178 rs3759035 rs12363614 rs7128076 rs10896073 rs9735063 rs1047464 rs7113428 rs2276132 rs10791837 rs747526 rs10896074 rs147982796 rs6591195 rs150188401 |
|  | rs754532 | -- |
|  | rs586921 | rs543952 rs622779 rs645571 rs1204011 rs1191716 rs1151510 rs1151511 rs1151514 rs9736731 rs559161 rs677350 rs610497 rs540584 rs507672 rs506873 rs1786171 rs1786172 rs522553 rs619701 rs55644043 rs1192184 rs590531 rs549334 rs502363 rs534201 rs35911045 rs616073 rs615646 rs1786174 rs11227377 rs11227378 rs75154345 rs632640 rs7933510 rs58833868 rs684546 rs548435 rs548495 rs668210 rs140250262 |
| *ACTL6A* | rs12631988 | rs12632721 rs73048716 rs10513761 rs4855090 rs4284980 rs4855091 rs4855092 rs16830587 rs55837066 rs2292907 rs2292905 rs7643532 rs78295396 rs16830600 rs74686416 rs4475040 rs149676657 rs35045240 rs12635617 rs61086485 rs73048727 rs79000506 rs73883565 rs10513762 rs2879605 rs113041545 rs3774260 rs113646525 rs73048730 rs111353757 rs114822697 rs6772319 rs59700512 rs7613710 rs11338795 rs6783120 rs6785779 rs113425680 rs4635704 rs112009668 rs4855097 rs3732998 rs16830629 rs73883552 rs2339844 rs73883571 rs113527645 rs58728627 rs4855098 rs12631863 rs4147787 rs6788205 rs4147792 |
|  | rs9816801 | rs13322072 rs10575925 rs9870205 rs7644051 rs12696481 rs7632781 rs199924842 rs6790272 rs6790039 rs201684609 rs9831673 rs4855096 rs3830546 rs6778219 |
|  | rs1132429 | rs6795642 rs9818068 rs71907893 rs199924842 rs9861172 rs7610384 rs7644051 rs7635877 rs13322072 rs35792644 rs6775032 rs12492220 rs5854839 rs113724918 rs7611557 rs7630511 rs9829395 rs6797961 rs4147788 rs9882051 rs13325172 rs7616758 rs7639131 rs2077675 rs2339846 rs10937007 rs12054439 rs10937008 |
| *CBX3* | rs10255851 | rs10233641 rs10277455 rs10247665 rs36032567 rs10236009 rs1025331 rs34876113 rs33998333 rs11971002 rs11974650 rs11983671 rs33998608 rs35060854 rs6974645 rs12700716 rs71521751 rs13227109 rs11486821 rs7803845 |
|  | rs2391265 | rs3757668 rs3757667 rs12672942 rs2059903 rs573090483 rs7806787 rs12672536 rs10265394 rs2074566 rs576084528 rs12113404 rs10230838 rs7798786 rs34181077 rs10951128 rs6461920 rs2115547 rs12669140 rs10275247 rs67976730 rs34954459 |
| *CFL1* | rs635375 | rs668356 rs2308219 rs656040 rs652021 rs4621 rs665306 rs667555 rs7125986 rs7947929 rs7947741 rs508548 rs1939212 rs13817 rs659857 rs545500 rs551294829 rs633877 rs538394 rs648732 rs630758 rs630755 rs630303 rs11332468 rs558114 rs60409156 rs679147 |
|  | rs11227332 | -- |
| *CRYAB* | rs2070894 | rs11603779 rs14133 rs1940391 rs7950926 rs12808601 rs12785809 rs3944619 rs11214037 rs11214036 rs4441050 rs71057102 rs7127010 rs10891297 rs57414412 rs7114617 rs75228430 rs11604704 rs4936834 rs11214029 rs11214028 rs34888817 rs35581942 rs12364361 rs12364358 rs7128862 rs7125417 rs1045282 rs11600652 rs10789851 rs10789850 rs7107213 rs7124696 rs4936804 rs7112886 rs7930208 rs72994380 rs34558891 rs4936800 rs11608193 |
|  | rs12284944 | rs10891299 rs10789852 rs4252591 rs57377042 rs57527690 rs10502152 rs11214043 rs34183951 rs7124407 rs7124433 rs11384304 rs5794769 rs1320664 rs34088139 |
|  | rs762550 | rs1940392 rs11214033 rs7114594 rs11214031 rs610747 rs2850245 rs634983 rs626243 |
|  | rs4252588 | -- |
| *NME1-2* | rs2318782 | rs11654411 rs34520637 rs2302254 rs11652807 rs11656090 rs11652793 rs368050652 rs34666604 rs12941230 rs11655050 rs35347299 rs8075231 rs8074416 rs11654212 rs11651032 rs11650103 rs2041296 rs8071647 rs6504696 rs62063055 rs35523133 rs12941552 rs2159359 rs62063023 rs3744660 |
|  | rs3760467 | rs78766181 rs4794206 rs1558254 rs118144056 rs74470375 rs75410762 rs74970602 rs2215290 rs56209259 rs147722568 rs2318784 |
|  | rs41524846 | rs545197307 rs71381337 |
|  | rs16949649 | rs3760468 rs7207370 rs36055638 rs34431255 rs34214448 rs11079945 rs11079944 rs12451567 rs12936304 rs12952884 rs35995934 rs11869537 rs571079699 rs28710472 |
|  | rs4605213 | rs8079266 rs545197307 rs2041297 |
|  | rs3760469 | rs11556835 rs7213328 rs28597917 rs12939771 rs35517913 rs28710472 rs9303575 rs9303574 |
|  | rs2318785 | -- |
|  | rs9303577 | rs9303578 rs34313614 rs8081088 rs59280351 rs1062386 rs3803886 rs537942439 rs11650690 rs8069453 rs7213683 rs34581166 rs10545166 rs8076679 rs12453388 rs9912882 rs11650030 rs11870136 rs35289073 rs12449426 rs17574235 rs28410310 rs12946936 rs9915099 rs9789063 rs11651110 |
|  | rs12942155 | rs12945077 rs59280351 rs9915554 rs12451629 rs532770255 rs7220360 rs8081088 rs8080943 rs4794210 rs34313614 rs9303578 rs9907636 rs35397252 rs9894098 rs537942439 rs11650690 rs929482 rs8069453 rs1062386 rs11079946 rs8079807 rs7213683 rs34581166 rs10545166 rs8076679 rs7222463 rs35987233 rs12453388 rs9912882 rs12453513 rs35172874 rs11650030 rs35269451 rs11870136 rs35289073 rs12449426 rs4273084 rs17574235 rs28410310 rs368640927 rs3815411 rs12946936 rs113570976 rs9915264 rs9915099 rs11656911 rs9789063 rs71300606 rs2079883 rs9910220 rs11651110 rs1860574 rs6416962 rs7207416 rs2215417 rs9905051 rs11079947 rs8076651 rs10550602 |
|  | rs12937424 | rs62063028 rs35292418 rs62063027 rs7208601 rs62063026 rs11868380 rs11869672 rs111481382 rs113131698 |
| *PRDX2* | rs10427027 | rs12151144 rs116813755 rs10421604 rs7507935 |
|  | rs1205171 | rs12609353 rs35858668 |
| *PRDX3* | rs1553850 | rs139394880 rs61876564 rs7080535 rs71016553 rs75475521 rs74566533 rs370067252 rs7078965 rs10886414 rs11198816 rs35439312 rs1247800 rs12412287 rs1108473 rs1108471 rs1108472 rs11594600 rs34061558 rs10510054 rs4752262 rs10886417 rs10749299 rs10886421 rs10787929 rs7923896 rs61876593 rs4752263 rs1980030 |
|  | rs3740562 | rs11198811 rs34060649 rs10886412 rs2271362 rs7768 rs10886413 rs1889742 rs7894367 rs1107942 rs35148933 rs10430698 rs146722239 rs7068409 rs7068525 rs7068698 rs7358070 rs10787925 rs10787926 rs35838962 rs7087504 rs10886418 rs11198819 rs7082425 rs7082346 rs10886419 |
|  | rs10886406 | rs2271362 |
| *PRDX4* | rs795493 | rs795494 rs795492 rs795491 rs473769 rs518329 rs552105 rs490513 rs1548734 rs2665364 rs497797 rs795485 rs795486 rs112139616 rs570823 rs11412736 rs547679 rs496635 rs795488 rs513573 rs513572 rs477204 rs478034 rs482702 rs6629721 rs512997 rs1704732 rs565208 rs693806 rs7063461 rs528683 rs528834 rs71817516 rs557914 rs496067 rs475171 rs525443 rs35647919 rs551029 rs557637 |
|  | rs528960 | rs497087 rs544971 rs35477162 rs795495 rs3838952 rs2532625 rs2109142 rs11094937 rs2665367 rs2159383 rs2665369 rs480691 rs34759783 rs476316 rs477914 rs7881384 rs5925869 rs2532626 rs518857 rs568062 rs812704 rs511120 rs140643281 rs572174 rs570466 rs569603 rs543050 rs2532630 rs2665371 rs578606 rs497967 rs552109 rs498728 rs6629723 rs2665363 |
|  | rs795489 | rs477233 rs576656 rs501655 rs503572 rs73209274 rs483169 rs527749 rs113389417 rs113128237 |
|  | rs3829725 | rs6629722 rs7056309 rs6629724 rs6627966 rs6629725 rs6629719 rs7052367 rs6627965 rs6629718 rs6627964 rs6629717 rs3795226 rs6629716 rs12384006 |
|  | rs564561 | rs478051 |
|  | rs3795220 | -- |
|  | rs525497 | -- |
|  | rs561909 | -- |
|  | rs499692 | rs5901737 rs35250716 rs474657 |
| *CAVIN1*  *(PTRF)* | rs7212299 | rs7210713 rs4796582 rs6503699 rs6416922 rs77390070 rs6416923 rs112865149 rs36116679 rs7207285 rs111383744 rs7503929 rs8074832 rs12950246 rs56387576 rs9906657 rs9909871 rs8079445 rs4796649 rs4796650 rs11422354 rs7502611 rs9709260 rs59371909 rs35972838 rs7221385 rs17841509 rs7217251 |
|  | rs7223784 | rs560819092 rs34967087 rs12937756 rs560117712 rs8068008 rs12936165 rs35272895 rs34610643 rs34156075 rs57307201 rs537482133 rs8064439 rs8081779 rs143185919 rs35470081 rs11871801 rs11867405 rs12938897 rs12938856 rs12948909 rs35510462 rs78594996 rs72823064 rs12938957 rs72029736 rs57203370 rs7213500 rs34527783 rs56001200 rs12936869 rs35261210 rs71367913 rs6503703 rs6503704 rs12951549 rs16649 rs12943498 rs12951640 rs35111218 rs142647634 rs36005199 rs375125601 rs36075544 rs34633576 rs149422595 rs34460267 rs1032070 rs34807589 |
|  | rs2883456 | rs11653437 rs709631 rs2128786 rs12453307 rs963987 rs963988 rs12150088 rs34154026 rs56364076 |
|  | rs1032072 | rs1032071 rs58747199 rs72823071 rs151285077 rs2062213 rs12603814 rs1968866 rs8071329 rs367572409 rs371774724 rs76681937 rs2883457 rs72626001 rs191327332 rs141580568 rs140220058 rs117512511 rs72823083 rs66774412 rs115341059 rs67560218 rs116971005 rs3760387 rs140294647 rs16967794 rs72626002 rs144967292 rs7224647 rs6416924 rs138251779 rs145289234 rs201794971 |
|  | rs9252 | -- |
|  | rs963986 | rs4365333 rs72823047 rs111550283 rs8078850 rs7222712 rs1129422 |
| *RPL28* | rs4806668 | rs3810168 rs3842412 rs3745272 rs1870074 rs73617860 rs73617855 |
|  | rs12981911 | rs10418020 |
|  | rs17700376 | -- |
|  | rs10425596 | rs11673539 |
|  | rs7255648 | rs7255657 rs56283327 rs78892292 rs200296013 rs142797565 rs78935016 rs74683747 rs78633024 rs75479073 rs11882786 rs3810167 rs12981704 rs141481594 rs148705147 rs76684947 |
| *TDP1* | rs9488 | rs17126538 rs10645574 rs61520219 rs112332158 rs35522230 rs11851131 rs10710186 rs8003210 rs8005676 rs2401866 rs67169479 rs7150605 rs11310221 |
|  | rs4143999 | rs3759710 rs2181599 rs28736874 rs34348197 rs12232164 rs146704566 rs17260562 rs10498624 |
|  | rs942190 | rs4904629 rs7147996 |
|  | rs7158356 | rs73324579 |
|  | rs1286927 | -- |
|  | rs119467003 | -- |
|  | rs2176075 | rs746752 rs8010627 rs35972056 |
|  | rs28365054 | rs143694338 rs34510655 rs75021372 rs73324561 rs34080955 rs34258347 rs11849310 rs11844949 rs75530159 rs35913426 rs35782158 rs35257587 rs34705515 rs113464893 rs34458659 rs113385610 rs17126508 rs34533254 rs34254473 rs113469707 rs144733015 rs58917724 rs57055713 rs36109363 rs35819811 rs79690576 rs80153327 rs78402658 rs34614135 rs35151660 rs34086909 rs34075974 rs35211637 rs36005472 rs35320469 rs8016664 rs8020872 rs8020897 rs113492506 rs11848062 rs17126510 rs34587290 rs35666995 rs35483543 rs17126513 rs35230069 rs34612773 rs35548006 rs35766668 rs17126514 rs58176524 rs35787421 rs7150748 rs7150652 rs7145132 rs75781929 rs34567445 rs34680032 rs35178878 rs75745280 rs7153551 rs35501816 rs535016899 rs8015261 rs8015277 rs8014111 rs8014295 rs34259367 rs8008263 rs11851399 rs11851415 rs35853694 rs34601269 rs8003068 rs12881272 rs12880397 rs12879968 rs34745798 rs34277635 rs35434877 rs34507901 rs59736885 rs12101181 rs112528751 rs73324588 rs12101164 rs1399742 rs78138961 rs148839615 rs11846794 rs75911102 rs113053120 rs112036275 rs35653575 rs6575100 rs8006050 rs34657096 rs6575098 rs7151882 rs75356978 rs74710063 rs7152455 rs17126520 rs34088863 rs35664925 rs17126522 rs36082908 rs17126485 rs59577883 rs112115508 rs111546207 rs111311830 rs77461748 rs10498623 rs8003693 rs17798962 rs9919910 rs34792766 rs151254830 rs4603488 rs17126527 rs77951315 rs111232232 rs78465035 rs58017722 rs74899305 rs61073988 rs79098962 rs10130775 rs10142587 rs10142377 rs10569454 rs201118162 rs11850323 rs11849387 rs10143622 rs149550699 |
|  | rs28365055 | rs28422802 rs2277518 rs60362204 |
|  | rs11851266 | rs34290480 rs8022306 rs7154092 rs2401863 rs12884144 |
| *GSTP1* | rs6591256 | rs6591255 rs7949587 rs7949394 rs17593068 rs11311625 rs36211088 rs36211089 rs2370141 rs1079719 rs7945035 rs2370143 rs7941648 rs762803 rs7941395 rs7927657 rs7940813 rs6591254 rs6591253 rs597297 rs6591252 rs6591251 rs4930444 rs7948911 rs6591249 rs7945927 rs7108426 rs7103713 rs35534379 rs12807265 rs10896181 rs55854833 rs4930436 rs7952081 rs7948823 rs7948478 rs75649061 rs61390583 rs11605586 rs7106675 rs7106423 rs34721562 rs34802338 rs6591245 rs4320958 rs1871036 rs200602459 rs555435772 rs6591244 rs7938563 rs57808037 |
|  | rs1695 | rs749174 rs1871042 rs4891 rs947895 rs7952248 rs6591258 rs7104580 rs11603735 rs4360710 rs7939505 rs6591259 rs7937159 rs7950707 rs4244823 rs11227845 rs7108149 rs7108038 rs12416994 rs11603991 rs7103632 rs12790798 rs12792496 rs35247127 rs12798262 rs572499532 rs4930450 rs4024254 rs12421329 rs12793832 rs1871043 |
|  | rs4147581 | rs694746 rs57025443 rs11278380 rs614080 rs625978 rs656652 rs674783 rs591434 rs658768 rs645653 rs11227837 rs1262166 rs666328 rs1269981 rs1254123 rs638140 rs10444362 rs55740781 rs684928 rs12225376 rs2508459 rs35840098 rs2008922 rs57387696 rs10896180 rs10896179 rs35829185 rs611663 rs598811 rs10791906 rs1148884 rs1148883 rs12365485 |
|  | rs7927381 | rs593055 rs612020 rs3082142 rs1254133 rs641544 rs643318 rs643317 rs137897748 rs655848 rs656798 rs627449 rs1871041 rs1254130 rs4930443 rs594784 rs685032 rs689317 rs688878 rs676653 rs675679 rs6591247 rs4930204 rs1254125 rs7128347 rs604908 rs669956 rs11405928 rs601531 rs604232 rs667014 rs615260 rs10667536 rs657003 rs644670 rs615270 rs612162 rs598398 rs694616 rs649149 rs661624 rs687316 rs34961815 rs584338 rs595262 rs629952 rs78166531 |
|  | rs1138272 | rs72934504 |
| *TIMP1* | rs2070584 | rs6609533 rs4898 rs6520279 rs5953060 rs5905615 rs34026683 |
|  | rs6520278 | rs12556415 rs5905614 rs5906434 rs6609534 rs55696512 rs111947105 |
|  | rs4824621 | rs4824622 rs62592125 rs150642282 rs5953061 rs5953062 rs5953063 rs5906437 rs9887335 rs5953066 rs4824424 rs4824623 |

Abbreviations: htSNPs, haplotype tagging SNPs; SNPs, single nucleotide polymorphisms. Associated SNPs are polymorphisms in strong linkage with the htSNP with r² > 0.80, as determined with the data of 1000 Genomes Phase 3 for the CEU population (Ensembl GRCh38 release 91 - December 2017).

**Supplementary Table 3.** htSNPs significantly (*P* < 0.1) associated with progression-free survival (PFS) in the Canadian cohort.

| **Genes** | **htSNPs** | **Base change** | **Model** | **HR_adj_ (95% CI)^a^** | ***P*** |
| --- | --- | --- | --- | --- | --- |
| *ACTL6A* | rs9816801 | G>C | dominant | 0.74 (0.53 - 1.05) | 0.095 |
| *CBX3* | rs2391265 | T>C | dominant | 1.48 (1.00 - 2.20) | 0.050 |
| *CFL1* | rs11227332 | A>G | recessive | 2.29 (1.05 - 5.01) | 0.038 |
| *GSTP1* | rs6591256 | A>G | dominant | 0.74 (0.53 - 1.04) | 0.080 |
| *GSTP1* | rs4147581 | C>G | dominant | 0.54 (0.37 - 0.80) | 0.002 |
| *MAPK9* | rs4362908 | T>C | dominant | 0.59 (0.41 - 0.86) | 0.006 |
| *MAPK9* | rs7724543 | A>G | dominant | 1.48 (1.02 - 2.15) | 0.039 |
| *MAPK9* | rs6896347 | G>A | dominant | 0.64 (0.43 - 0.96) | 0.030 |
| *MAPK9* | rs11956696 | A>G | dominant | 1.42 (1.01 - 2.01) | 0.047 |
| *MAPK9* | rs4147385 | G>A | recessive | 1.88 (0.95 - 3.72) | 0.071 |
| *MAPK9* | rs7445806 | C>T | dominant | 0.69 (0.49 - 0.97) | 0.031 |
| *NME1-2* | rs9303577 | A>C | recessive | 1.43 (0.96 - 2.12) | 0.079 |
| *NME1-2* | rs2318785 | A>G | dominant | 1.37 (0.97 - 1.94) | 0.077 |
| *PARP1* | rs3219110 | T>C | recessive | 1.39 (0.95 - 2.03) | 0.087 |
| *PRDX4* | rs795493 | A>G | dominant | 0.65 (0.47 - 0.92) | 0.014 |
| *PRDX4* | rs525497 | C>G | dominant | 1.35 (0.95 - 1.92) | 0.092 |
| *PRDX4* | rs528960 | A>G | recessive | 0.50 (0.24 - 1.02) | 0.057 |
| *PRDX4* | rs795489 | A>G | recessive | 1.44 (1.02 - 2.05) | 0.039 |
| *PRDX4* | rs561909 | C>A | recessive | 1.56 (0.94 - 2.59) | 0.089 |
| *RPL28* | rs4806668 | G>T | recessive | 3.23 (1.29 - 8.14) | 0.013 |
| *RPL28* | rs10425596 | T>C | recessive | 1.91 (0.93 - 3.93) | 0.080 |

^a^ Adjusted for age and cotreatment. Abbreviations: CI, confidence interval; HR_adj_, adjusted hazard ratio; htSNPs, haplotype tagging single nucleotide polymorphisms.

**Supplementary Table 4.** htSNPs significantly (*P* < 0.1) associated with overall survival (OS) in the Canadian cohort.

| **Genes** | **htSNPs** | **Base change** | **Model** | **HR_adj_ (95% CI)^a^** | ***P*** |
| --- | --- | --- | --- | --- | --- |
| *ACTL6A* | rs9816801 | G>C | dominant | 0.70 (0.49 - 1.01) | 0.053 |
| *CBX3* | rs2391265 | T>C | dominant | 1.48 (0.99 - 2.20) | 0.054 |
| *MAPK9* | rs4362908 | T>C | dominant | 0.58 (0.40 - 0.85) | 0.005 |
| *MAPK9* | rs7724543 | A>G | dominant | 1.41 (0.97 - 2.06) | 0.073 |
| *MAPK9* | rs12522876 | C>A | dominant | 0.69 (0.45 - 1.05) | 0.082 |
| *MAPK9* | rs6896347 | G>A | dominant | 0.58 (0.38 - 0.89) | 0.013 |
| *MAPK9* | rs11956696 | A>G | recessive | 2.03 (0.88 - 4.70) | 0.099 |
| *MAPK9* | rs4147385 | G>A | recessive | 1.99 (1.00 - 3.96) | 0.050 |
| *NME1-2* | rs9303577 | A>C | recessive | 1.62 (1.07 - 2.44) | 0.023 |
| *PARP1* | rs3219123 | G>A | dominant | 0.58 (0.31 - 1.07) | 0.083 |
| *PRDX3* | rs1553850 | T>A | dominant | 1.38 (0.96 - 1.98) | 0.085 |
| *PRDX4* | rs795493 | A>G | dominant | 0.71 (0.51 - 1.01) | 0.053 |
| *CAVIN1*  *(PTRF)* | rs7223784 | A>C | recessive | 2.90 (1.33 - 6.32) | 0.008 |
| *RPL28* | rs4806668 | G>T | recessive | 3.09 (1.10 - 8.65) | 0.032 |

^a^ Adjusted for age and cotreatment. Abbreviations: CI, confidence interval; HR_adj_, adjusted hazard ratio; htSNPs, haplotype tagging single nucleotide polymorphisms.

**Supplementary Table 5.** The marker rs4806668G>T and its linked SNPs are significantly associated to the expression of *RPL28* in the transverse colon (GTEx dataset), but not the expression of the nearby *TMEM238* gene.

| **Gene** | **SNP** | ***P*-value** |
| --- | --- | --- |
| *RPL28* | rs4806668G>T | **0.0067** |
| *RPL28* | rs3810168T>C | **0.00057** |
| *RPL28* | rs3745272C>G | **0.013** |
| *RPL28* | rs73617860T>G | **0.0082** |
| *RPL28* | rs73617855T>A | **0.0035** |
| *TMEM238* | rs4806668G>T | 0.92 |
| *TMEM238* | rs3810168T>C | 0.79 |
| *TMEM238* | rs3745272C>G | 0.89 |
| *TMEM238* | rs73617860T>G | 0.87 |
| *TMEM238* | rs73617855T>A | 0.90 |

Data were not available for rs3842412-/15-mer and rs1870074A>G. *P* < 0.05 are in bold.

**Supplementary References**

1 Hoskins, J. M. *et al.* Irinotecan pharmacogenetics: influence of pharmacodynamic genes. *Clin Cancer Res* **14**, 1788-1796, (2008).

2 Allen, W. L. *et al.* A systems biology approach identifies SART1 as a novel determinant of both 5-fluorouracil and SN38 drug resistance in colorectal cancer. *Mol Cancer Ther* **11**, 119-131, (2012).

3 Davidson, D., Wang, Y., Aloyz, R. & Panasci, L. The PARP inhibitor ABT-888 synergizes irinotecan treatment of colon cancer cell lines. *Invest New Drugs* **31**, 461-468, (2013).

4 Peng, X. C. *et al.* Proteomic analysis of cell lines to identify the irinotecan resistance proteins. *J Biosci* **35**, 557-564, (2010).

5 Gong, F. M. *et al.* Comparative proteomic analysis of irinotecan-sensitive colorectal carcinoma cell line and its chemoresistant counterpart. *Anticancer Drugs* **22**, 500-506, (2011).

6 Pommier, Y., Leo, E., Zhang, H. & Marchand, C. DNA topoisomerases and their poisoning by anticancer and antibacterial drugs. *Chem Biol* **17**, 421-433, (2010).

7 Kweekel, D. M. *et al.* GSTP1 Ile105Val polymorphism correlates with progression-free survival in MCRC patients treated with or without irinotecan: a study of the Dutch Colorectal Cancer Group. *Br J Cancer* **99**, 1316-1321, (2008).

8 Braun, M. S. *et al.* Association of molecular markers with toxicity outcomes in a randomized trial of chemotherapy for advanced colorectal cancer: the FOCUS trial. *J Clin Oncol* **27**, 5519-5528, (2009).

9 Sorensen, N. M. *et al.* TIMP-1 is significantly associated with objective response and survival in metastatic colorectal cancer patients receiving combination of irinotecan, 5-fluorouracil, and folinic acid. *Clin Cancer Res* **13**, 4117-4122, (2007).

10 Bystrom, P. *et al.* Evaluation of predictive markers for patients with advanced colorectal cancer. *Acta Oncol* **51**, 849-859, (2012).
